# A new ataxia-telangiectasia mutation in an 11-year-old female

**DOI:** 10.1007/s00251-017-0983-9

**Published:** 2017-05-09

**Authors:** Esmaeil Mortaz, Sayed Mehran Marashian, Hosseinali Ghaffaripour, Mohammad Varahram, Payam Mehrian, Atosa Dorudinia, Johan Garssen, Ian M. Adcock, Malcolm Taylor, Seyed Alireza Mahdaviani

**Affiliations:** 1 Clinical Tuberculosis and Epidemiology Research Center, National Research Institute of Tuberculosis and Lung Diseases (NRITLD), Shahid Beheshti University of Medical Sciences, Tehran, Iran; 2 Division of Pharmacology, Utrecht Institute for Pharmaceutical Sciences, Faculty of Science, Utrecht University, Utrecht, The Netherlands; 3 Chronic Respiratory Diseases Research Center, National Research Institute of Tuberculosis and Lung Diseases (NRITLD), Shahid Beheshti University of Medical Sciences, Tehran, Iran; 4 Pediatric Respiratory Diseases Research Center, National Research Institute of Tuberculosis and Lung Diseases, NRITLD, Shahid Beheshti University of Medical Sciences, Tehran, Iran; 5 Mycobacteriology Research Center, National Research Institute of Tuberculosis and Lung Diseases (NRITLD), Masih Daneshvari Hospital, Shahid Beheshti University of Medical Sciences, Tehran, Iran; 6 Nutricia Research Centre for Specialized Nutrition, Utrecht, Netherlands; 7 Cell and Molecular Biology Group, Airways Disease Section, Faculty of Medicine, National Heart and Lung Institute, Imperial College London, London, UK; 8 Priority Research Centre for Healthy Lungs, Hunter Medical Research Institute, The University of Newcastle, Newcastle, NSW Australia; 9 Institute of Cancer and Genomic Sciences, University of Birmingham, Edgbaston, Birmingham, UK

**Keywords:** A-t, Mutation, Female

## Abstract

Ataxia-telangiectasia (A-T), a rare inherited disorder, usually affects the nervous and immune systems, and occasionally other organs. A-T is associated mainly with mutations in the ataxia telangiectasia mutated (ATM) gene, which encodes a protein kinase that has a major role in the cellular response to DNA damage. We report here a novel ATM mutation (c.3244_3245insG; p.His1082fs) in an 11-year old female. This subject presented with typical features, with the addition of chest manifestations including mediastinal lymphadenopathy and diffuse bilateral micronodular infiltration of the lungs, along with a high EBV titer. The subject died as a result of rapid B-cell lymphoma progression before chemotherapy could be initiated. This case highlights the need for the rapid diagnosis of A-T mutations and the detection of associated life-threatening outcomes such as cancers.

## Introduction

Ataxia-telangiectasia (A-T), a rare inherited disorder, usually affects the nervous and immune systems, and occasionally other organs. It is characterized by progressive difficulty with coordinating movements (ataxia) beginning in early childhood, usually before age five. Affected children typically develop difficulty in walking and experience imbalance and loss of hand coordination, involuntary jerking movements (chorea), muscle twitches (myoclonus), and disturbances in nerve function (neuropathy). Small clusters of enlarged blood vessels called telangiectasia occur in the eyes and on the skin surface, which are also characteristic of A-T. Characteristic immunological features of A-T include lymphopenia and Ig deficiencies, and patients also show abnormal vascular development, radiation hypersensitivity, and increased incidence of cancer (Matei et al. 2006; Mortaz et al. 2016; Riballo et al. 2004).

A-T is associated chiefly with the ataxia telangiectasia mutated (ATM) gene, which encodes a protein kinase that has a major role in the cellular response to DNA damage. ATM mutations crucially lead to a defective ability to repair DNA double-strand breaks (DSBs) as well as to blunted immune cell development in some cases (Mortaz et al. 2016). ATM was cloned in 1995 (Savitsky et al. 1995a); it is located on human chromosome 11 (11q22.3) and is composed of 69 exons spread across 150 kb of genomic DNA (Gatti et al. 1988). Numerous mutations in the ATM gene have been identified in classical A-T and in some patients with the variant form of A-T (Concannan and Gatti 1997). The majority of the published mutations in the ATM gene are truncating, although missense substitutions and in-frame-deletions have also been reported (Baumer et al. 1996; Savitsky et al. 1995b; Stankovic et al. 1998). The exon-intron structure of the ATM gene has been fully elucidated (Uziel et al. 1996), and the complete ATM sequence is available, enabling the mutation scanning of genomic DNA (Platzer et al. 1997).

The current report describes an examined patient and her relatives with symptoms and signs of A-T, who were either referred to us or invited by our group to be investigated for the presence of novel A-T mutations.

### Case presentation and results

An 11-year girl was referred to the Masih Daneshvari Hospital, a university referral center for pulmonary diseases (Tehran, Iran), complaining of cough, dyspnea, weight loss, motor regression, and fever for 5 months prior to admission. Her medical history revealed a cerebral palsy when she was 9 years old concomitant with a fever of unknown origin (FUO). In terms of family history, her mother had died due to breast cancer, and her parents had a familial marriage background.

### Physical examination and laboratory finding

At admission, the girl showed ataxia, nystagmus, atrophic tonsils, and auxiliary fever (38.5 °C), telangiectasia in her eyes, and an abnormal gait in addition to bilateral crackles in chest auscultation. Her erythrocyte sedimentation rate (ESR) was 36 mm/h, thyroid assessment and liver function tests gave values in the normal range, and the viral load for CMV and EBV was 301 and 480,000 copies/ml, respectively. Serum concentrations for IgG, IgA, IgM, and IgE were 440, 30, 100, and 1 mg/dl, respectively, which were all below normal levels. The isohemagglutinin titer ratio was 1/256. Assessment of antibody response showed 0.2 for tetanus and 0.5 for diphtheria toxoid vaccines. Bone marrow aspiration was performed and normal findings were concluded. Smear and culture from sputum for tuberculosis was negative.

Serum lactate dehydrogenase (LDH) was high at 644 Units/l (normal range 135–376 U/l) as were serum triglycerides (TG), which were 294 mg/dl (normal <150 mg/dl). Serum fibrinogen (200 mg/dl) was at the bottom of the normal range (200–400 mg/dl). Flow cytometry of peripheral venous blood gave monocyte cell proportions as CD3+ T cells (48%), CD4+ T cells (12%), CD19+ cells (3.34%), and CD56+ natural killer cells (32%).

An elevated serum α-feto-protein level was seen.

A-T was considered to be a strong diagnosis based on the patient’s clinical manifestation and was treated with irregular courses of intravenous immunoglobulin (IVIG) (500 g/dl) and antibiotics, including vancomycin and ceftazidime.

### Imaging studies

**Fig. 1:**
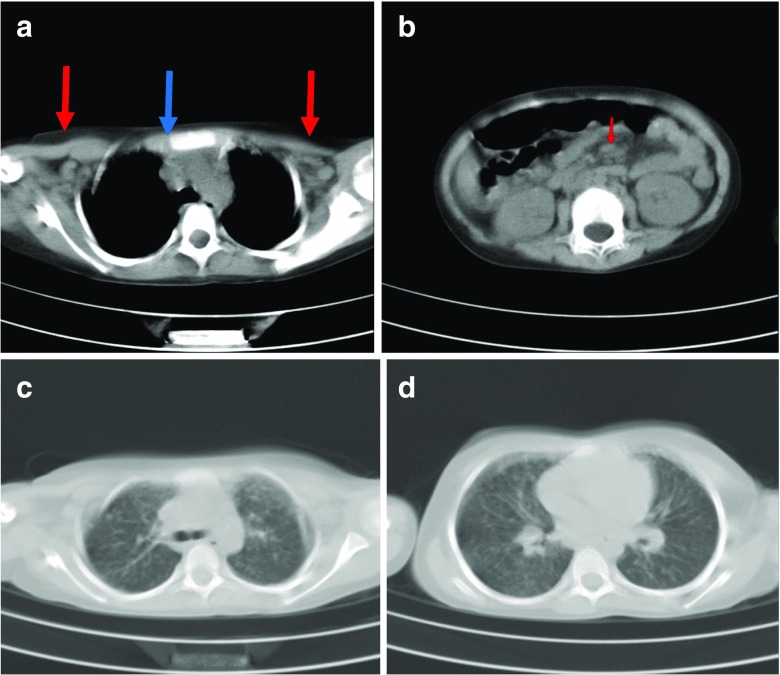
CT scan of lungs. **a** Axial chest CT scan (mediastinal window). Bilateral axillary (*red arrows*) and mediastinal lymphadenopathies (*blue arrow*). **b** Axial abdominal CT scan. Mesenteric lymphadenopathy (*red arrow*). **c** Axial chest CT scan without contrast. **d** Bilateral micronodular opacities along with reticulonodular infiltrations are seen

**Fig. 2:**
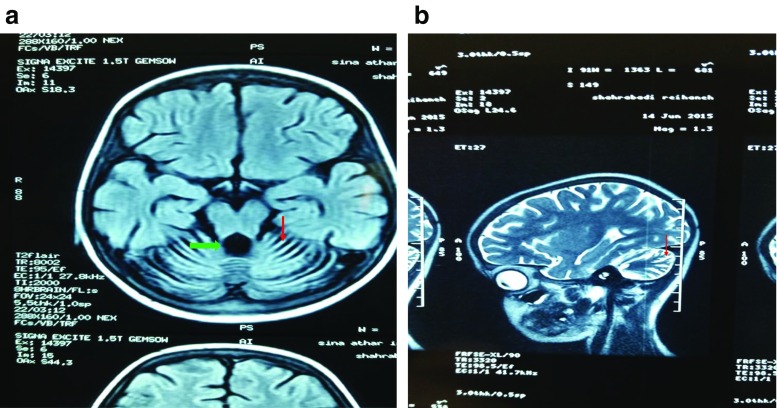
Brain MRI. Axial T1-weighted (**a**) and sagittal T2-weighted (**b**) brain MRI. The diffuse cerebellar volume loss with enlargement of cerebellar sulci (*red arrow*), vermian atrophy and compensatory enlargement of the fourth ventricle (*green arrow*)

A chest CT scan demonstrated bilateral axillary (Fig. 1a) and mediastinal (Fig. 1b) adenopathies along with diffuse bilateral micronodular infiltration in lungs. Abdominal and pelvic CT scan and ultrasound revealed enlarged mesenteric, para aortic, aortocaval and bilateral inguinal lymph nodes (Fig. 1c, d). Brain MRI (axial T1 weighted (Fig. 2a) and sagittal T2 weighted (Fig. 2b)) images demonstrated diffuse cerebellar atrophy with enlarged cerebellar sulci (Fig. 2a, b red arrow) and compensatory dilation of fourth ventricle (Fig. 2a green arrow).

**Fig. 3:**
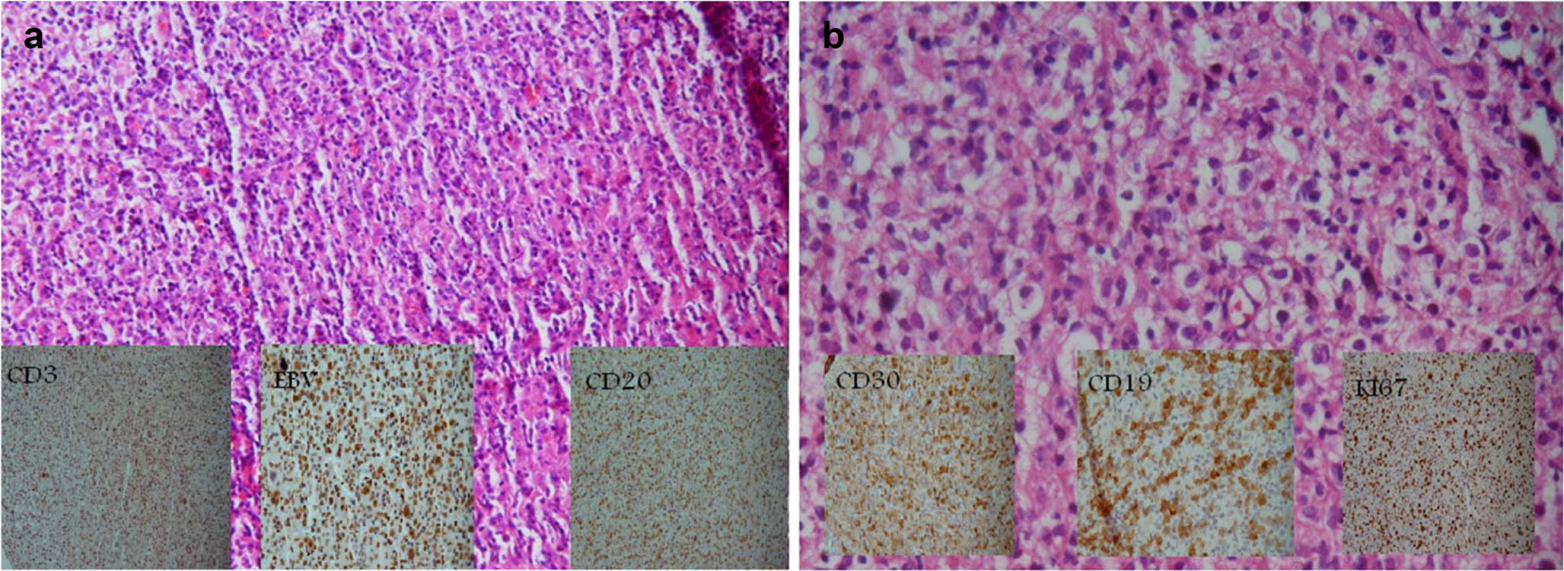
Supraclavicular lymph node staining. **a** Totally effaced lymph node with infiltration of scattered large immunoblasts intermixed with smaller bland-looking lymphoid cells that are mixed T cells (*left inset image*, CD3+) and B cells (*right inset image*, CD20+). Cells are positive for EBV infection (*central inset image*). B cells are polyclonal due to the EBV-associated lymphoproliferative disorder. **b** After 5 months, the right cervical lymph node shows mixed infiltration of lymphocyte plasma cells and histiocytes intermixed with large cells with lobular and nucleolated nuclei that are stained positive for the CD30 (*left inset image*), CD19 (*central inset image*) and negative for CD20, CD3, CD43, CD23, CD5, and CD10. KIi67 (*left inset image*) was positive for 70% of neoplastic cells

H&E staining of the neck lymph node biopsy showed polyclonal lymphoproliferative disease, which was suggestive of EBV-related lymphadenopathy (Fig. Fig. 3a). Rituximab 375 mg/m^2^ IV infusion was administered to the patient, resulting in an improvement in her general condition. Treatment was continued for 4 weeks but the fever reappeared when the treatment stopped. Immunologic analysis at this point revealed that the subject was negative for serum human herpes virus 8; LDH = 879 U/L, white blood cell count = 1100 (80% polymorphonuclear leukocytes, 8% lymphocytes), metHb = 7.3 g/dl, alanine aminotransferase = 35,000 IU/l, C-reactive protein = 48, and ESR = 22.

Rituximab 375 mg/m^2^ IV infusion was prescribed again for an additional 4 weeks. Cervical lymphadenopathy reappeared, and a mixed infiltration of different immune cells as well as large atypical lymphocytes was detected. IHC analysis detected CD30+, CD20−, CD19, and LCA+ cells in addition to finding a B cell lymphoma (Fig. Fig. 3b). Immunodefficiency-related B cell lymphoproliferative disorder, consistent with diffuse large B cell lymphoma, was reported. The patient died unexpectedly from fever and respiratory distress before her chemotherapy started.

The patient and two of her relatives who had similar manifestations were invited to be checked for their genetic features.

### DNA analysis

Whole-blood DNA was extracted and transferred to the Institute of Cancer and Genomic Sciences, Vincent Drive, Edgbaston, Birmingham, UK. DNA from the patient and her two relatives was sequenced, and mutations in the ATM region in all three patients in the homozygous state were identified to perfectly designate: “c.3244_3245insG; p.His1082fs.” This single-base insertion causes a frameshift truncating mutation and results in instability and loss of the ATM protein from both alleles. This was a new mutation that had not been previously described. The patient also had the polymorphism c.8786+8A>C.

## Discussion

This case report demonstrates a new A-T mutation in a subject with typical disease presentation associated with a high EBV titer. Our subject died as the onset of B cell lymphoma symptoms were detected before chemotherapy could be initiated. This case highlights the need for rapid diagnosis of A-T mutations and the detection of associated life-threatening outcomes such as cancers and EBV-associated LPD (lymphoproliferative disorders).

A-T is a heterogeneous autosomal recessive disease with progressive multisystem characteristics mainly associated with progressive neurodegenerative changes, in addition to cerebellar ataxia and variable presentation of immunodeficiency. These kinds of involvements of the lungs, especially adenopathies along with diffuse bilateral micronodular infiltration, have not previously been reported. A-T is also associated with a susceptibility to sinopulmonary infection and predisposes patients to malignancy (Mannaa 2016; Rothblum-Oviatt et al. 2016; Bhatt et al. 2015). A-T has an incidence of 1/40,000–1/300,000 subjects worldwide, depending upon the region (Rothblum-Oviatt et al. 2016; Sedgewick et al. 1972; Swift et al. 1986). The genetic basis of A-T was described in 1995 and caused 35 deaths in the USA in 2005–2007. From among these 35, 17 died from respiratory problems and 11 from malignancy.

Pulmonary manifestations of A-T are usually seen in 25% of patients, and these range from a weak cough and wheezing to recurrent pneumonia, bronchiectasis, and even interstitial lung disease (ILD) (Rothblum-Oviatt et al. 2016; Bhatt et al. 2015; Crawford et al. 2006). The lung involvement in A-T involves several mechanisms including bulbar dysfunction and immune defects; some risk factors such as cigarette smoke, pollution, poor nutrition, and female gender (particularly in cystic fibrosis cases) play crucial roles (Bhatt et al. 2015). Cancer occurs in a similar percentage (25%) of A-T patients during their lifetime (Bhatt et al. 2015; Reiman et al. 2011; Suarez et al. 2015). The most common types of malignancy in A-T patients are lymphoma and leukemia, especially in those <16–20 years of age. However, adults with A-T are more prone to both lymphoid and solid tumors (Rothblum-Oviatt et al. 2016; Bhatt et al. 2015). The mortality rate of cancer in A-T patients is 22%.

Carriers with a single mutated copy of the ATM gene are generally healthy, although a reduced lifespan due to different types of malignancy was revealed in this group in a meta-analysis. In contrast, the increased risk of breast cancer in A-T female patients is up to 2.3-fold greater than in the normal population (Rothblum-Oviatt et al. 2016; van Os et al. 2016; Hollestelle et al. 2010; Economopoulou et al. 2015; Thompson et al. 2005; Renwick et al. 2006). This suggests that an early diagnosis of A-T noting earlier manifestations such as neurologic and swallowing problems as well as respiratory recurrent infections may help prevent death from A-T-associated cancers (Rothblum-Oviatt et al. 2016).

The pronounced changes in serum Ig levels in our patient, in addition to increased serum LDH and some neurologic and pulmonary symptoms and signs, may together be used as indicators to assess the risk of cancer and could facilitate beginning chemotherapy earlier in other patients. Evidence also indicates that the main risk factor with regard to mortality is the possibility of respiratory tract infections in patients with hypomorphic mutations in ATM, although those with null mutations in “class A” disease have an increased risk of cancer at a younger age (Rothblum-Oviatt et al. 2016; Bhatt et al. 2015).

In some cases of atypical A-T with reduced severity, cancer detection may precede the diagnosis of A-T (Bielorai et al. 2013; Yanofsky et al. 2009). These cancers may also differ from those observed in classical A-T: for example, a case report involving a 6-year-old child with A-T with Hodgkin lymphoma in comparison to the more common incidence of non-Hodgkin lymphoma (Mannaa 2016; Niedobitek et al. 2001).The humoral immunodeficiency in A-T patients can affect serum IgA and/or IgG2 levels (Staples et al. 2008). However, 10% of patients have increased levels of serum IgM concentrations with a deficiency of IgG and IgA (Noordzij et al. 2009). Nevertheless, the levels of IgM in the current patient were within the normal range (Nowak-Wegrzyn et al. 2004). Interestingly, the virology data indicated that a high EBV viral load possibly triggered development of the malignancy in this patient (Niedobitek et al. 2001; Yukio Sakiyama et al. 1984). This suggests that detection of EBV in A-T patients may indicate the future development of malignancy in this disorder. In relation to the new mutation that we found in our case, Liu and co-workers reported two new mutations in ATM including c.8911 C>T and c.7141-7151 delAATGGAAAAAT in three early-onset A-T subjects, which are comparable with that reported here (c.3244_3245insG;p.His1082fs) (Liu et al. 2016).

To conclude, we report here a new mutation in A-T, which presents with classical symptoms and predisposes to cancers; our subject died just as B cell lymphoma was detected and before chemotherapy could be started. This case highlights the importance of early diagnosis of A-T and the rapid detection of life-threatening outcomes such as cancers and EBV-associated LPD.
